# The role of EZH2 in tumour progression

**DOI:** 10.1038/bjc.2011.551

**Published:** 2011-12-20

**Authors:** C-J Chang, M-C Hung

**Affiliations:** 1 Department of Molecular and Cellular Oncology, The University of Texas MD Anderson Cancer Center, Houston, TX 77030, USA; 2 The Center for Molecular Medicine and Graduate Institute of Cancer Biology, China Medical University and Hospital, Taichung 404, Taiwan

**Keywords:** EZH2, Polycomb proteins, histone methyltransferase

## Abstract

Accumulated evidence shows that EZH2 is deregulated in a wide range of cancer types, and it has a crucial role in stem cell maintenance and tumour development. Therefore, blocking EZH2 expression or activity may represent a promising strategy for anticancer treatment. In this review, we address the current understanding of the mechanisms underlying EZH2 regulation alongside the function of EZH2 gene targets that are involved in cancer progression. Finally, we will describe cancer therapies that target EZH2 or its downstream cascades, which could potentially reverse the oncogenic and stemness properties of the tumour cells to suppress cancer progression and recurrence.

EZH2 is the catalytic core protein in the Polycomb Repressor Complex 2 (PRC2), which catalyses the trimethylation of histone3 lysine27 (H3K27) and mediates gene silencing of the target genes that are involved in fundamental cellular processes, such as cell fate decision, cell cycle regulation, senescence, cell differentiation and cancer (Sauvageau and Sauvageau, 2010). Recent findings implicate that EZH2 deregulation could be an important driver of tumour development and progression, and that inactivation of EZH2 may be therapeutically effective in many cancers.

## Tumourigenic role of EZH2

EZH2 is highly expressed in a wide range of cancer types, including breast, prostate, bladder, colon, lung, pancreatic cancer, sarcoma and lymphomas Overexpression of EZH2 is often correlated with advanced stages of human cancer progression and poor prognosis (Sauvageau and Sauvageau, 2010). Enforced expression of EZH2 in cell lines shows increased proliferation and oncogenic capacity. Overexpressing EZH2 in mammary epithelial cells of the tumourigenic mouse model using mammary tumour virus long-terminal repeat (MMTV-EZH2) leads to epithelial hyperplasia phenotype (Li *et al*, 2009). Furthermore, somatic *EZH2* mutations and deletions are found in 22% of germinal-centre diffuse large B-cell lymphomas, 7% of follicular lymphomas, and 12–23% of patients with myelodysplasic and myeloproliferative disorders. Most of the mutations are known to inactivate the enzymatic activity of the PRC2 complex (measured by the methylation status of an unmodified histone peptide substrate), which firstly seems to contradict the oncogenic properties of EZH2 (Piunti and Pasini, 2011). For example, in lymphoma, a heterozygous missense mutation frequently occurs at amino acid Y641, within the SET domain of EZH2. It was observed that wild-type EZH2 displays highest catalytic activity for the first mono-methylation of H3K27, but relatively weak capability for the subsequent di- and tri-methylation. On the contrary, the Y641 mutant displays limited ability in catalysing mono-methylation, but acquires higher catalytic efficiency for the subsequent reactions. Unsurprisingly, it was shown that the mutant Y641 allele is always found associated with a wild-type allele (heterozygous) in B-cell lymphoma cells. Heterozygous Y641 mutant, thus, can work in conjunction with wild-type EZH2 to augment H3K27 methylation, which may be functionally equivalent to EZH2 overexpression (Chase and Cross, 2011). Similarly, mutations reported in some epigenetic regulators that are functionally related to EZH2 are also shown to be involved in oncogenesis. For example, inactivating mutations of an H3K27 demethylase, ubiquitously transcribed tetratricopeptide repeat gene on X chromosome (UTX), recurrently occur in a wide range of malignancies, which may be functionally equivalent to EZH2 overexpression (Chase and Cross, 2011).

In contrast to the mutations seen in lymphoma, EZH2 mutations in myeloid neoplasms in SET domain are mostly nonsense and stop codon mutations, and thus are deficient in their histone methyltransferase activity (Chase and Cross, 2011). As either activating or inactivating mutations of EZH2 can be associated with certain types of cancer, the complex role of EZH2 mutants in cancer needs to be further investigated in terms of whether these EZH2 mutants exert differential regulation of a specific cohort of target genes that contribute to oncogenesis.

## EZH2 function and recruitment on chromatin

When the PRC2 complex is recruited to chromatin, the histone methyltransferase EZH2 catalyses the trimethylation of the lysine 27 of histone H3 (H3K27me3), which leads to subsequent recruitment of the PRC1 complex that monoubiquitylates the lysine 119 of histone H2A (H2AK119ub1) to prevent RNA polymerase II-dependent transcriptional elongation and consolidate transcriptional repression (Sauvageau and Sauvageau, 2010). Several reports suggest that EZH2 directly interacts with DNA methyltransferases (DNMT1, DNMT3A, DNMT3B) and that EZH2 is necessary for the maintenance of DNA methylation and stable repression of specific genes, including many tumour suppressors (Sauvageau and Sauvageau, 2010).

In *Drosophila*, Polycomb group proteins (PcGs) are recruited to specific DNA sequences, Polycomb response elements (PREs). The vertebrate murine PRE was identified as a palindromic double PHO (mammalian ortholog as YY1)-binding site. In human embryonic stem cells, a potential 1.8-kb PRE that contains YY1 binding sites was recently found between the HOXD11 and HOXD12 loci, where YY1, PRC1 and PRC2 components are recruited to this PRE (Morey and Helin, 2010).

Some studies show that DNA binding factors are also involved in recruitment of PcGs to specific target genes. For example, JARID2 is able to bind to PcG target genes and the interaction is required to recruit PRC2 to these target genes in ES cells. Furthermore, in acute promyelocytic leukaemia, a fusion oncoprotein, promyelocytic leukaemia-retinoic acid receptor alpha (PML-RARα), recruits PRC2, nucleosome-remodelling complex and DNMTs to the target promoters, whereas knocking down PRC2 component results in promoter reactivation and granulocytic differentiation (Villa *et al*, 2007). Similarly, two other leukaemic fusion proteins, PLZF-RARα and TMPRSS2-ERG (Boukarabila *et al*, 2009; Yu *et al*, 2010), can also recruit PRC2 and PRC1 to specific target genes, suggesting that oncogenes may target PcGs as a key step for carcinogenesis.

It was indicated that 20% of the large intervening noncoding RNAs (lincRNA) are associated with PRC2. Among these lincRNAs, HOTAIR is found to recruit the PRC2 complex to the Hox loci. HOTAIR is often overexpressed in the metastatic breast tumours (Gupta *et al*, 2010). Loss of HOTAIR impairs cancer cell invasiveness, whereas ectopic expression of HOTAIR relocalises PRC2 complex to bind to the target genes that are signified in embryonic fibroblasts (Gupta *et al*, 2010). These data suggest a critical role of lincRNAs in the regulation of PRC2/EZH2 recruitment to specific target genes that contribute to cancer progression.

## EZH2 linking stem cells to cancer

Numerous studies indicate the role of EZH2 in the maintenance of self-renewal of adult and ES cells. A genome-wide integrative analysis shows that a significant subset of the PRC2 target genes in aggressive prostate cancer are also targets of PRC2 in embryonic stem (ES) cells, and their repression in tumours is associated with poor prognosis (Yu *et al*, 2007). A shared gene expression signature enriched in PRC2 target genes and Oct4/Sox2/Nanog target genes further reveals a direct link between poorly differentiated human tumour cells and ES cells (Piunti and Pasini, 2011). Overexpression of EZH2 has also been linked to cancer initiation and progression. It is speculated that during oncogenesis, a plastic chromatin state could progress towards permanent silencing through acquisition of DNA methylation or Polycomb-mediated histone methylation. As EZH2 expression is expected to be low in differentiated tissue cells, overexpression of EZH2 could reinforce or promote dormant progenitors or more differentiated cells to an aggressive stem cell-like state. Furthermore, high-grade tumours are enriched with a high content of cancer stem cells, and it was proposed that a more aggressive secondary cancer stem/progenitor cell population may arise from a primary cancer stem cell population through acquisition of additional genetic mutations that deregulate cancer stem/progenitor homeostasis and drive cancer progression (Visvader and Lindeman, 2008). The involvement of EZH2 overexpression in cancer stem cells and cancer progression was consolidated by recent finding, which elucidates that EZH2 contributing not only to cancer stem cell formation but also to expansion of an aggressive cancer stem cell population that promotes cancer progression (Chang *et al*, 2011). In this study, Chang *et al.* identify a mechanism by which EZH2 expression-mediated downregulation of DNA damage repair leads to accumulation of recurrent RAF1 gene amplification in breast tumour-initiating cells (BTICs), which activates downstream signalling to promote BTIC expansion in aggravated breast cancer.

## Regulation of EZH2 in cancer

Accumulated evidence indicates that EZH2 can be regulated in different types of human cancers at transcriptional, post-transcriptional and post-translational levels. It was shown that transcription factors E2Fs bind to the PRC2- *Ezh2* and *Eed* promoters and transactivate their expression, which is required for E2F mediated-cell proliferation (Bracken *et al*, 2003) (Table 1). EZH2 expression can also be transcriptionally activated by a fusion oncoprotein EWS-FLI1 in Ewing's sarcoma, and induced EZH2 expression has a key role in endothelial/neuroectodermal differentiation and tumour growth (Richter *et al*, 2009; Table 1). On the contrary, SNF5, a chromatin-remodelling subunit, was shown to directly repress *Ezh2* transcription, and deregulated EZH2 expression is required for SNF5-deficiency-induced lymphoma (Wilson *et al*, 2010; Table 1).

The microenvironment of solid tumours contains regions of poor oxygenation as a result of hypoxia. It is noteworthy that hypoxia/HIF1*α* activation is associated with high-grade basal breast cancer and poor prognosis. A recent study further reveals that EZH2 expression in the BTIC population is particularly enhanced by hypoxia through HIF1α-mediated transactivation, which in turn promotes the expansion of BTICs and cancer progression (Chang *et al*, 2011; Table 1).

In addition to transcriptional regulation, *Ezh2* transcript is known to be regulated by several micro-RNAs. For example, miR-26a binds to and inhibits *Ezh2* transcript expression in lymphoma (Sander *et al*, 2008;Table 1). Of note, miR-101, which is frequently lost in metastatic prostate tumours, targets the 3′UTR of *Ezh2* mRNA and promotes its degradation (Varambally *et al*, 2008; Table 1).

EZH2 can also be modulated by a variety of post-translational modifications. EZH2 is phosphorylated by AKT on Ser21, which results in decreased PRC2 histone methyltransferase (HMT) activity and contributes to tumour development (Cha *et al*, 2005; Table 1). Recent discoveries from several groups indicate that EZH2 is regulated by cell-cycle-dependent signalling through phosphorylation at Thr350/487 by CDK1 or CDK2 (Chen *et al*, 2010; Wei *et al*, 2011; Table 1). It was shown that CDK1 phosphorylation of EZH2 at Thr487 leads to reduced HMT activity due to disrupted interaction of EZH2 with other PRC2 components (Wei *et al*, 2011; Table 1), whereas another report indicates that phosphorylation of EZH2 at Thr345/487 promotes EZH2 ubiquitination and subsequent degradation (Wu and Zhang, 2011; Table 1). However, there are other studies demonstrating that CDK1 phosphorylation of EZH2 promotes recruitment of EZH2 to specific target loci (Kaneko *et al*, 2010; Table 1), and that inhibiting CDK1 could enhance EZH2 target gene expression, potentially by increasing the binding of EZH2 to lincRNAs (please also refer to the previous section). These functional discrepancies of CDK1-mediated EZH2 phosphorylation may be the result of target gene- or cell type-specific manners and are expected to be resolved by future studies.

In addition, exposure to tobacco smoke condensate (TSC) induces recruitment of EZH2 to Wnt-antagonist *Dkk1* promoter, leading to activated oncogenic Wnt signalling in lung cancer cells (Hussain *et al*, 2009; Table 1). However, the mechanism by which TSC promotes EZH2 association to *Dkk1* promoter is unclear and also needs to be further investigated.

## Direct EZH2 targets in cancer

Accumulated evidence shows that EZH2 contributes to various aspects of cancer by regulating a myriad of target genes. It was shown that EZH2-containing PRC2 transcriptionally represses cell cycle suppressor INK-ARF to drive cell cycle progression, prevent cell senescence and also exhaustion of stem/cancer stem cells (Bracken *et al.*, 2007; Table 2). EZH2 can also inhibit astroglial differentiation and promote glioma tumourigenecity by repressing BMP Receptor1-Beta (BMPR1B) expression and BMPR1B-mediated differentiation signalling (Lee *et al*, 2008; Table 2).

In addition, EZH2 promotes epithelial–mesenchymal transition (EMT), a process that is associated with cancer progression and metastasis, by interacting with transcription factor SNAIL1 and suppressing expression of epithelial marker E-cadherin (*CDH1*) (Cao *et al*, 2008; Table 2). EZH2 was also shown to trigger metastasis directly through epigenetic silencing of a RasGAP gene, Disabled Homolog2-Interacting Protein (DAB2IP). This gene functions as a signalling scaffold that coordinately regulates Ras and NFκB. By suppressing DAB2IP, EZH2 activates Ras and NF-*κ*B to promote prostate tumour initiation and metastasis (Min *et al*, 2010; Table 2). Integrative genomics analysis also reveals another critical EZH2 target gene, adrenergic receptor beta-2 (ADRB2), in metastatic prostate cancer. Inhibition of ADRB2 by EZH2 induces cell invasiveness and transformation of prostate epithelial cells (Yu *et al*, 2007; Table 2).

Moreover, EZH2 is implicated in promoting tumour angiogenesis. It shows that VEGF, which stimulates angiogenesis, can upregulate E2F1/3 transcription factors to transactivate EZH2 expression. Increased EZH2 expression by VEGF silences expression of a negative regulator of angiogenesis, Vasohibin1 (VASH1), and subsequently enhances angiogenesis (Lu *et al*, 2010; Table 2).

A recent study further identified that under hypoxia insult, induced EZH2 expression downregulates DNA damage repair protein RAD51 expression, which leads to genomic aberrations, such as RAF1 gene amplification, to promote RAF1-ERK-*β*-catenin signalling and expansion of BTICs (Chang *et al*, 2011; Table 2). This finding further reveals that AZD6244, a clinical trial drug that inhibits RAF1-ERK signalling, is effective in preventing breast cancer progression by eliminating BTICs (Chang *et al*, 2011; Table 2).

Together, these studies indicate that EZH2 has an essential and multi-faceted role in cancer. Blocking EZH2 expression or activity may represent a promising strategy for anticancer treatment targeting tumour cells, tumour endothelial cells and tumour stem cells.

## Potential cancer therapeutics targeting Polycomb function or downstream signalling

It is known that EZH2 is crucial in stem cell maintenance and tumour development. Reducing EZH2 expression using siRNA or treatment of a small-molecule-S-adenosylhomocysteine hydrolase inhibitor 3-deazaneplanocin (DZNep) that inhibits methyltransferases and induces degradation of EZH2 was shown to result in cell growth inhibition and reduced tumour formation in various cancers (Piunti and Pasini, 2011). For example, disruption of EZH2 by DZNep or by short-hairpin RNAs (shRNAs) was shown to significantly impair self-renewal and the tumour-initiating capacity of glioblastoma cancer stem cells (Suva *et al*, 2009) or ovarian cancer stem cell-like populations (Rizzo *et al*, 2011). Downregulation of EZH2 by shRNAs *in vivo* significantly decreased breast xenograft tumour growth and improved survival (Gonzalez *et al*, 2009). Similarly, DZNep was shown to be effective in inhibiting prostate cancer cell growth and its anti-tumour activity is in part mediated by suppressing the tumourigenic potential of the prostate cancer stem cell (Crea *et al*, 2011). However, the therapeutic value of DZNep needs to be further assessed, as it is not a specific EZH2 inhibitor; methylation of other histone lysines and arginines can be globally inhibited by DZNep.

In addition to the direct regulation of EZH2 activity, cancer therapy that blocks EZH2-mediated oncogenic signalling is warranted. A recent study illustrates that EZH2 can induce RAF1-ERK-*β*-catenin signalling, which in turn enhances the survival and proliferation of BTICs (Chang *et al*, 2011). This finding further reveals a previously unidentified therapeutic effect of RAF1-ERK signalling inhibitors in preventing breast cancer progression by eliminating BTICs and elucidates important clinical implications.

## Future Perspectives

As mentioned above, EZH2 can be regulated through various mechanisms. Also, it is plausible that modulation of these EZH2-regulatory mechanisms could significantly impact EZH2 activity and be therapeutically effective in many cancers. For example, it was shown that EZH2 expression is highly enhanced by hypoxia through HIF1α-mediated transactivation in the BTICs and contributes to cancer progression (Chang *et al*, 2011). Hypoxia inducible factor (HIF) inhibitors have been used in clinical trials and have shown marked effects in inhibiting tumour growth (Semenza, 2003). As suggested in Chang *et al.*, HIF inhibitors could also potentially be effective in suppressing EZH2 oncogenic function in tumour stem cells to prevent cancer recurrence. In addition, it was found that aberrant activation of CDK1/2 decreases tumour suppressor gene *DAB2IP* expression and contributes to the aggressiveness of cancer cells by phosphorylating EZH2 at Thr350 (Chen *et al*, 2010). Thus, using CDK1/2 inhibitors to dephosphorylate EZH2 is also likely to disable EZH2-mediated oncogenesis.

Furthermore, it was found that, similar to the effects of knocking down EZH2, overexpression of miR-101 inhibits proliferation and invasiveness of cancer cells *in vitro*. It will be appealing to test whether expressing miR-101 using microRNA therapy can cause therapeutic effects *in vivo* as microRNA therapy has been exploited in preclinical and clinical trials as a potential cancer treatment regimen (ClinicalTrials.gov).

However, previous work demonstrated that conditional inactivation of Ezh2 in mouse adult stem cells (from hematopoietic, brain or pancreatic tissues) could yield minor defects in normal organ development or functions (Abdel-Wahab and Levine, 2010). Therefore, administering EZH2 inhibitors by a tumour-specific delivery system may be necessary to avoid the side effects in normal (stem) cells. Furthermore, better characterising and specifically blocking EZH2-mediated oncogenic targets/signalling pathways can be more efficient and effective.

Together, understanding the regulatory mechanisms of EZH2 and the function of EZH2 gene targets will shed light on the development of novel cancer treatments targeting EZH2/Polycomb mediated-tumourigenesis to suppress oncogenic and stemness-associated pathways, which will ultimately achieve the goal of cancer prevention and remission.

## Figures and Tables

**Table 1:** Regulators of EZH2 expression or activity in cancer

**Regulator**	**Working mechanism**	**Cancer type**	**Reference**
E2F	Transcriptional activation	Non-specific	Bracken *et al*, 2003
EWS-FLI1	Transcriptional activation	Ewing tumours	Richter *et al*, 2009
SNF5	Chromatin repression	Malignant rhabdoid tumours/lymphoma	Wilson *et al*, 2010
HIF1α	Transcriptional activation	Breast	Chang *et al*, 2011
miR-26a/miR-101	Repression of EZH2 transcript	Lymphoma/prostate	Sander *et al*, 2008; Varambally *et al*, 2008
AKT	Phosphorylation, decreased EZH2 HMT activity	Breast	Cha *et al*, 2005
CDK1/2	Phosphorylation, altered EZH2 recruitment to targets	Breast	Chen *et al*, 2010; Kaneko *et al*, 2010; Wei *et al*, 2011

Abbreviation: HMT=Histone methyltransferase.

**Table 2:** Direct EZH2 targets and their function in cancer

**Target**	**Target function**	**Cancer type**	**Reference**
DKK1	Wnt inhibitor	Lung	Hussain *et al*, 2009
INK/ARF	Cell cycle and senescence regulator	Non-specific	Bracken *et al*, 2003
BMPR1B	Pro-differentiation	Glioma	Lee *et al*, 2008
CDH1	EMT regulator	Prostate	Cao *et al*, 2008
DAB2IP	Ras and NF-*κ*B inhibitor	Prostate	Min *et al*, 2010
ADRB2	Metastasis inhibitor	Prostate	Yu *et al*, 2007
VASH1	Angiogenesis inhibitor	Ovarian	Lu *et al*, 2010
RAD51	DNA damage repair	Breast	Zeidler, 2005; Chang *et al*, 2011

Abbreviation: NF-κB= nuclear factor kappa-light-chain enhancer of activated B cells.
